# Genetic Factors Associated with a Poor Outcome in Head and Neck Cancer Patients Receiving Definitive Chemoradiotherapy

**DOI:** 10.3390/cancers11040445

**Published:** 2019-03-29

**Authors:** David M. Vossen, Caroline V.M. Verhagen, Martijn van der Heijden, Paul B.M. Essers, Harry Bartelink, Marcel Verheij, Lodewyk F.A. Wessels, Michiel W.M. van den Brekel, Conchita Vens

**Affiliations:** 1 Division of Cell Biology, The Netherlands Cancer Institute, 1066 CX Amsterdam, The Netherlands; d.vossen@nki.nl (D.M.V.); cverhagen@rijnstate.nl (C.V.M.V.); ma.vd.heijden@nki.nl (M.v.d.H.); m.verheij@nki.nl (M.V.); 2 Department of Head and Neck Oncology and Surgery, The Netherlands Cancer Institute, 1066 CX Amsterdam, The Netherlands; p.essers@nki.nl (P.B.M.E.); m.vd.brekel@nki.nl (M.W.M.v.d.B.); 3 Department of Radiation Oncology, The Netherlands Cancer Institute, 1066 CX Amsterdam, The Netherlands; h.bartelink@nki.nl; 4 Division of Molecular Carcinogenesis, The Netherlands Cancer Institute, 1066 CX Amsterdam, The Netherlands; l.wessels@nki.nl; 5 Department of EEMCS, Delft University of Technology, 2600 AA Delft, The Netherlands; 6 Institute of Phonetic Sciences, University of Amsterdam, 1012 WX Amsterdam, The Netherlands; 7 Department of Oral and Maxillofacial Surgery, Academic Medical Center, 1105 AZ Amsterdam, The Netherlands

**Keywords:** head and neck squamous cell carcinoma, pharyngeal neoplasms, genomics, DNA sequence analysis, mutation, chemoradiotherapy, prognosis

## Abstract

About half of advanced stage head and neck squamous cell carcinoma (HNSCC) patients can be cured by chemoradiotherapy. Patient outcome may be partially determined by the genetic alterations in HNSCC, rendering these alterations promising candidate prognostic factors and/or therapeutic targets. However, their relevance in patient outcome prognosis remains to be assessed in patients that receive standard-of-care chemoradiotherapy. We therefore tested whether frequent genetic alterations were associated with progression free survival (PFS) in advanced stage HNSCC patients who were uniformly treated with definitive platinum-based chemoradiotherapy. To this end, we performed targeted DNA sequencing on frozen pre-treatment tumor biopsy material from 77 patients with advanced stage oro- and hypopharyngeal carcinoma. This provided somatic point mutation and copy number aberration data of 556 genes. The most frequently mutated genes, *TP53* (62%), *CCND1* (51%), *CDKN2A* (30%) and *PIK3CA* (21%), were not associated with PFS. However, co-occurring *CCND1* and *CDKN2A* mutations were associated with short PFS (HR 2.24, *p* = 0.028) in HPV-negative tumors. Furthermore, tumor mutational burden (sum of somatic point mutations) showed a trend towards decreased PFS (HR 1.9, *p* = 0.089), and chromosomal instability (CIN) was associated with shorter PFS (HR 2.3, *p* = 0.023), independent of HPV status. Our results show that tumor mutational burden, CIN markers, and co-occurring *CCND1* and *CDKN2A* mutations are associated with chemoradiotherapy outcomes in advanced stage oro- and hypopharyngeal HNSCC patients, thereby highlighting their prognostic potential. Given their poor prognosis association and link to biological targets, they may also identify patients for novel targeted therapies and immunotherapies.

## 1. Introduction

Advanced stage head and neck squamous cell carcinoma (HNSCC) has a poor prognosis. Chemoradiotherapy cures about half of all patients with advanced HNSCC. Allowing the preservation of organ function, it has become the preferred treatment option for these cancers [1]. Patients with a poor prognosis under such standard treatment may be eligible for increased surveillance and/or alternative treatments if they could be identified upfront. However, the current TNM staging [2] based prognosis is very limited in this regard and novel prognostic biomarkers are therefore urgently needed. 

Tumor growth and treatment responses are largely determined by tumor intrinsic genetics. Genomic features therefore hold the promise of providing potential treatment response biomarkers. Frequent alterations are of particular interest for this purpose. Previous studies identified commonly mutated genes through large comprehensive DNA sequencing efforts and genetic analyses, thereby revealing the “driver genes” in HNSCC [3,4,5]. Their prognostic value is currently under investigation [6,7,8,9,10,11,12]. Pairs of co-occurring mutated genes can also hold prognostic information [13], and this can point to a functional link between the events. This can also apply across different types of genetic features in tumors; e.g., mutations in mismatch repair genes are linked to a high mutational burden [14] and homologous recombination repair gene mutations to overall gene copy number alterations (CNAs) [15].

DNA crosslink repair defect analyses in a large panel of patient-derived HNSCC cell lines revealed that HNSCC are frequently characterized by DNA repair (DR) defects [16]. Potentially disrupting mutations were found in multiple genes of this pathway in both patient tumors and cell lines. We also found that such mutations are associated with a poor outcome in HNSCC patients treated with chemoradiotherapy [16]. While DR provides cellular drug and radiation resistance through repair, it also governs genomic stability. Defects in DNA repair thus promote genomic instability, thereby enabling cancer cells to acquire new genetic traits, including those necessary to become therapy resistant. Indeed, the degree of a tumor’s genomic instability or DNA repair status is associated with poor prognosis in cancer and can therefore be of prognostic value [17,18]. In a previous study we showed that genomic instability measures vary between tumors of the oral cavity, larynx, and pharynx [15]. Measurements of genomic instability and correlates of DNA repair defects vary, but markers thereof can be obtained with genomic profiling. The total number of somatic point mutations (SPMs) in tumor DNA (“mutational burden”), alterations in ploidy, and/or the presence of chromosomal alterations—gains or losses—termed chromosomal instability (CIN) are possible markers of such factors [19]. 

Important questions, as to the prognostic value of the genomics in HNSCC, remain. Many studies focused solely on individual alterations. Even though frequent in HNSCC [4,5], the joint prognostic value of SPMs and CNAs in HNSCC has also not been assessed yet. In addition, previously studied cohorts often comprised tumors of various HNSCC sites. Patients were treated dissimilarly with a bias towards surgical resection of the tumor in most studies. However, HNSCC tumors from different anatomic sites have distinct genomic profiles [15]. Consequently, it remains to be determined which genetic markers are prognostic in specific sites treated with a uniform and contemporary modality. Importantly, since the large TCGA HNSCC study incorporated solely samples from resected HNSCC and included mostly oral cavity tumor specimens, oro- and hypopharyngeal carcinoma patients treated with standard-of-care definitive chemoradiotherapy are either not included or largely underrepresented in most current studies.

We therefore aimed to identify prognostic genetic factors of advanced stage pharyngeal squamous cell carcinoma treated with chemoradiotherapy. We performed a retrospective study based on pre-treatment tumor biopsies from 77 advanced stage HNSCC patients that all received cisplatin-based chemoradiation. Targeted DNA sequencing of these tumor biopsies was performed to identify gene mutations, and SPMs and CNAs were determined. These data were used to address the following questions: Are (1) mutated genes or co-occurring mutated genes, (2) mutational burden and/or (3) CIN markers associated with progression free survival (PFS)?

## 2. Results

### 2.1. Patient Characteristics and Sequencing

In order to assess the potential prognostic value of frequent genetic factors, we genetically characterized 77 oro- and hypopharyngeal carcinoma samples (patient characteristics shown in Table 1). The median PFS in this cohort was 5.8 years (95% CI: 4.5–8.9). As reported previously in similar patient cohorts, PFS was significantly linked to tumor site and HPV status (Table 1, Supplementary Materials: Figure S1 and Table S1). Tumor site and HPV status were therefore consistently included as independent variables in multivariable Cox models when testing for associations between genetic factors and PFS. Non-silent SPMs and CNAs were determined on targeted sequencing data of 556 genes (Supplementary Materials: Table S2) to identify gene mutations, deletions, and amplifications. In total, 416 non-silent SPMs were found in the sequenced genes, comprising mostly missense mutations (~80%, Supplementary Materials: Table S3). 

In addition to individual gene mutations, we used the sum of all SPMs (including silent) in all sequenced genes in a tumor sample as a measure of its overall mutational burden. The median mutational burden was 5 per sample or 2.8 mutations per Mb (Supplementary Materials: Figure S2), a value comparable to previous findings by whole exome sequencing in HNSCC [20]. CNA analyses on all targeted and sequenced genes provided ploidy estimates that served as a CIN marker. A total of 42% of the samples were classified as CIN+ according to this analysis. Together, our HNSCC data confirmed the mutational burden and chromosomal instability rich features that are characteristic to pharyngeal HNSCC [15].

### 2.2. “HNSCC Driver Genes”

**Figure 1:**
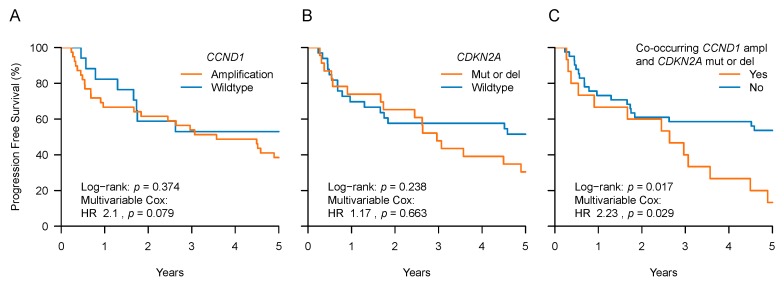
Progression free survival (PFS) according to genetic alterations in *CCND1* and *CDKN2A*. Kaplan–Meier curves of advanced stage pharyngeal HNSCC patients classified according to tumor *CCND1* and *CDKN2A* status: (**A**) *CCND1* amplification versus wildtype. (**B**) *CDKN2A* mutation or deletion versus wildtype. (**C**) *CCND1* amplification co-occurring with *CDKN2A* mutation or deletion, versus wildtype for one or either of these two genes. In-figure legends of A–B state hazard ratios (HR) and corresponding *p*-values from multivariable Cox models that were fitted on all (*n* = 77) samples and included the tested genetic factor, HPV status, and tumor site. In-figure legend of C states the hazard ratio (HR) and corresponding *p*-value from a multivariable Cox model that was fitted on these HPV-negative samples only (*n* = 56) and included the tested genetic factor and tumor site. Note, all graphs display Kaplan–Meier curves of HPV-negative samples only (*n* = 56) with the corresponding log–rank test.

First, we aimed to investigate the association of known “HNSCC driver genes”, as determined by Iorio et al. and based on SPM and CNA data [4], with PFS (see Methods and Supplementary Materials: Tables S4 and S5). For these PFS association analyses and consistent with the Iorio et al. identification criteria, only the genetic alteration type by which a gene was originally detected as a potential driver was considered [4]. For example, *CDKN2A* was identified as a potential HNSCC driver gene by its frequent presence of SPMs and deletions but not by amplifications. Amplifications were therefore not considered for *CDKN2A* sample classification, but SPMs and deletions were. The PFS association analyses were further restricted to frequently mutated genes only, a valuable requirement for any potential prognostic factor. In this study we therefore restricted the analyses to those genes which were mutated in the tumors of at least 10 patients, as this also allowed us to adequately fit a regression model [21,22]. Of all genes, the tumor suppressor gene *TP53*, the cell cycle regulators *CCND1* and *CDKN2A*, and the growth signaling gene *PIK3CA* met these criteria (Table 2). Notably, *CCND1* and *CDKN2A* alterations were only found in the HPV-negative tumor samples. We then used multivariable Cox models (with HPV and tumor site, see above) to investigate whether mutations in these genes were associated with PFS. Only in the *CCND1* analyses we found a trend towards shorter PFS for carriers of tumors with such amplifications (Table 2 and Supplementary Materials: Figure S3). This was the case regardless of whether HPV-positive samples were included or not (Table 2 and Figure 1A,B).

We next investigated pairs of co-occurring mutated genes. Tumors were classified as positive, in which both genes were mutated, and the rest as negative. Three pairs of co-occurring mutated genes were tested after applying the same restriction as above, of minimal 10 patients for each classification (Table S6). We found that patients with tumors harboring co-occurring *CCND1* mutations (amplifications) and *CDKN2A* mutations (SPMs and deletions) had shorter PFS than the rest (multivariable Cox: HR 2.24, 95% CI: 1.09–4.61, *p* = 0.028) (Supplementary Materials: Figure S3C).

In HPV-positive tumors, no mutations were detected in the cell cycle regulators *CCND1* or *CDKN2A*. This is consistent with the lack of a requirement for such mutations since the HPV viral protein E7 affects cell cycle regulation. Importantly, the prognostic significance of co-occurring *CCND1* and *CDKN2A* mutations remained even after excluding HPV-positive tumors (multivariable Cox: *p* = 0.029, Figure 1C).

Taken together, we tested recurrently mutated “HNSCC driver genes” for an association with PFS. Although none of the individual genes reached significance, the co-occurrence of *CCND1* and *CDKN2A* mutations was associated with shorter PFS in our patient dataset.

### 2.3. CCND1 and CDKN2A Expression and Patient Outcome

**Figure 2:**
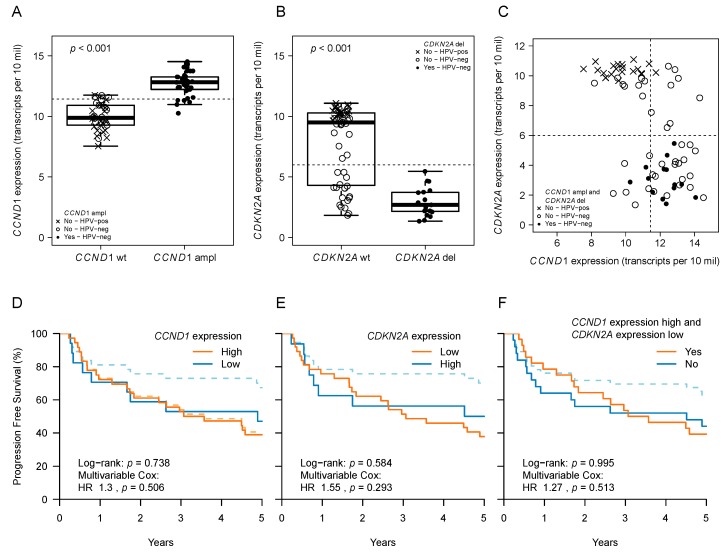
*CCND1* and *CDKN2A* expression and patient outcome. (**A**) *CCND1* expression in tumors with (‘ampl’) and without (‘wt’) *CCND1* amplification. (**B**) *CDKN2A* expression in tumors with (‘del) and without (‘wt’) *CDKN2A* deletion. (**C**) *CDKN2A* against *CCND1* expression per tumor sample, colored according to the presence (filled circles) or absence (open circles) of a co-occurring *CCND1* amplification and *CDKN2A* deletion. Dashed lines demarcate the median expression, according to which the samples received a high or low expression classification in D–F. (**D**–**F**) Kaplan–Meier curves of progression free survival (PFS) according to *CCND1* and *CDKN2A* expression status. Dashed lines represent all samples; solid lines represent HPV-negative samples only. Note, orange lines overlap in E–F. In-figure legends in D–F state hazard ratios (HR) and corresponding *p*-values from multivariable Cox models that were fitted on all (*n* = 77) samples and included the tested genetic factor, HPV status, and tumor site. In-figure stated log–rank tests correspond to the analyses in HPV-negative samples only.

Of the 15 tumors that carried co-occurring *CCND1* and *CDKN2A* mutations, all carried a *CCND1* amplification, 1/15 had a *CDKN2A* frameshift SPM, and 14/15 a *CDKN2A* deletion. Gene copy number aberrations were thus the primary gene alteration in this HNSCC subgroup that displayed significantly shorter PFS. We reasoned that these CNAs might be reflected in RNA expression changes which could therefore be used as a prognostic biomarker. To test whether this was feasible, we analyzed RNA-seq data available for 74 of the 77 tumors. Indeed, we found that *CDKN2A* deletion was associated with low *CDKN2A* expression (*p* < 0.001) and *CCND1* amplification with high *CCND1* expression (*p* < 0.001) (Figure 2A–C). However, *CCND1* and *CDKN2A* expression could not accurately discriminate tumors with co-occurring *CCND1* amplifications and *CDKN2A* deletions (Figure 2C).

We then classified tumors into low and high expression groups using the median expression as the threshold value. High *CCND1* expression and *CDKN2A* expression showed an association with poor prognosis in univariate models based on all samples (log–rank *p* = 0.032 and *p* = 0.01, respectively). However, this was due to HPV-positive tumors and hence lost in the multivariable analyses (Figure 2D,E). Tested as a numeric (continuous) variable, as opposed to the median split, expression was not significantly associated with PFS either (data not shown). A co-occurrence analysis based on the median expression classifications did not reveal an association with PFS either (Figure 2F). 

Taken together, the prognostic value of *CCND1* and *CDKN2A* was evident in the gene mutational analysis but not in the expression analyses. In fact, RNA expression analyses were unable to discriminate between *CDKN2A* wildtype and deleted *CDKN2A* in the HPV-negative samples (Figure 2B). At this stage, it is difficult to assess whether this may be a consequence of technical sensitivity issues due to stromal contamination.

### 2.4. Mutational Burden and CIN

We next aimed to investigate the prognostic value of genomic instability and DR defect related markers, as these are common in HNSCC [15]. Limited by the lack of whole exome or genome sequencing data, we used the estimates of mutational burden and CIN determined by ploidy estimates as described in the Materials and Methods Section. We find that in comparison to HPV-positive tumors, HPV-negative tumors had a higher mutational burden (*p* = 0.006) and were more often classified as CIN+ (*p* < 0.001) (Table 3). 

**Figure 3:**
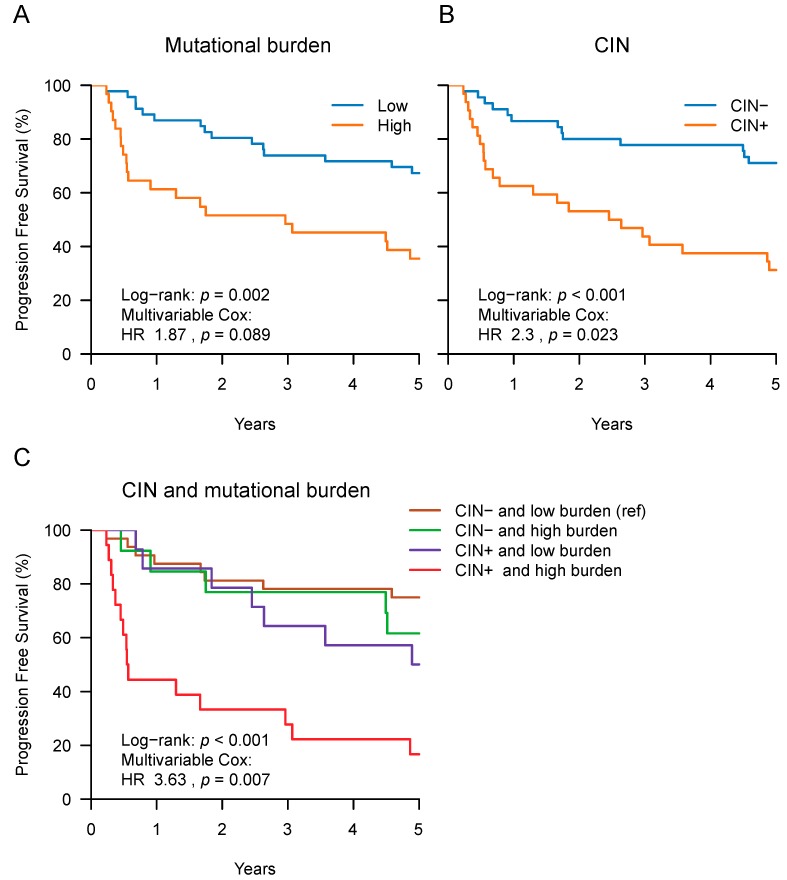
Progression Free Survival (PFS) according to mutational burden and chromosomal instability. Kaplan–Meier curves of PFS after patient classification by the following genomic factors in their tumors: (**A**) High or low mutational burden, (**B**) presence (CIN+) or absence (CIN-) of chromosomal instability markers (CIN) and (**C**) mutational burden and CIN. In-figure legends state hazard ratios (HR) and *p*-values from multivariable Cox models that included the tested genomic factor, HPV status, and tumor site. The HR shown in (**C**) refers to patients that have tumors with a high mutational burden and that show presence of CIN in their tumors, compared to those with a low mutational burden and absence of CIN.

We then tested whether mutational burden and CIN were associated with PFS using multivariable Cox models. When divided into “high” and “low mutational burden” groups according to the median mutational burden value, patients with a high tumor mutational burden had a trend towards shorter PFS (multivariable Cox with tumor site and HPV: HR 1.87, 95% CI: 0.91–3.86, *p* = 0.089) (Figure 3A and Table 3). CIN was associated with PFS as patients with CIN+ tumors had shorter PFS than those without (multivariable Cox: HR 2.3, 95% CI: 1.12–4.71, *p* = 0.023) (Figure 3B and Table 3). 

Although CIN+ tumors had a “high mutational burden” classification more often than tumors without CIN (56% versus 29%, *p* = 0.02), this did not always co-occur. Importantly, when included into the same multivariable model, with tumor site and HPV status as co-variables, CIN remained significant, and mutational burden showed a trend (*p* = 0.019 and *p* = 0.07, respectively). In this model, both CIN+ (HR 2.35, 95% CI: 1.15–4.81) and high mutational burden (HR 1.91, 95% CI: 0.95–3.86) were associated with shorter PFS. Importantly, it was the patients with tumors classified by both genetic features, a CIN+ and a high mutational burden, who had the shortest PFS (multivariable Cox: HR 3.63, 95% CI: 1.41–9.31, *p* = 0.007) (Figure 3C). 

Next, we combined all three prognostic genetic factors to assess whether they were independent predictors of PFS. We first tested for possible associations of co-occurring *CCND1* and *CDKN2A* mutations with mutational burden and CIN in the samples and found those alterations to be more prevalent in the HPV-negative samples (Supplementary Materials: Figure S4). Given the association of these genetic alterations with each other and with HPV status (Supplementary Materials: Figure S4), it is worthwhile to assess their independent prognostic value in a multivariable analysis. In fact, the association of high mutational burden and CIN with short PFS remained as a trend in multivariable Cox models that combined mutational burden, CIN, co-occurring *CCND1* and *CDKN2A* mutations, tumor site, and HPV status (with *p* = 0.079 and *p* = 0.059, respectively).

Taken together, we found that the presence of CIN was associated with short PFS in our cohort (in an HPV-status independent manner). Furthermore, the combined presence of CIN and a high mutational burden marked patients with a particular poor prognosis.

## 3. Discussion

We found that a high mutational burden showed a trend towards poor prognosis and that CIN/aneuploidy was associated with short PFS in our cohort of advanced stage oro- and hypopharynx carcinoma patients treated with chemoradiotherapy. A high mutational burden has been linked to poor prognosis in surgically resected HNSCC [8] and other cancer types [13,23]. Similarly, the association between CIN and poor prognosis has been demonstrated in various cancer types [24,25,26], including surgically resected HNSCC [27]. Mutant-allele tumor heterogeneity (MATH), a mutation-based measure of genetic heterogeneity, was found to be associated with poor prognosis in resected HNSCC [28]. Our findings thus confirm this poor prognostic pattern also for HNSCC patients who received chemoradiotherapy. They suggest that mutational burden and CIN are potential biomarkers in advanced stage HNSCC treated with chemoradiotherapy. DNA repair defects can cause genomic instability [29,30] and can therefore correlate with measurements thereof, such as mutational burden or CIN. Our results are therefore consistent with our previous reports demonstrating the presence of DNA repair defects in HNSCC and its association with poor prognosis [16]. Although therapeutic exploitation of CIN is currently not feasible in the clinic, strategies to do so are under investigation [31,32]. Tumor DNA repair defects on the other hand can be exploited by the use of DNA repair inhibitors [33].

The mutation frequencies in the “HNSCC driver genes”, defined by Iorio et al., that were observed in our study are consistent with similar HNSCC reports [3,8,10,11,34]. However, not all “HNSCC driver genes” [3,4] were sequenced in this patient cohort. The prognostic value of the remaining genes still needs to be determined for patients treated with chemoradiotherapy in future research. Individually, none of the mutated genes that were analyzed was associated with PFS in our cohort. This was despite, or because of, stringent statistical requirements for analysis (minimum of 10 patients per group). An improved outcome of patients with *NOTCH1*-mutated tumors was reported in a similar cohort [11]. Due to the limitation in cohort size, this finding was based on two events (deaths) in 12 patients with *NOTCH1*-mutated tumors. *NOTCH1* was part of our target genes but did not meet the frequency criteria in our cohort. Yet, when testing this reported association in our cohort, we did not observe an association between *NOTCH1* mutations status and PFS (data not shown). It should be noted, however, that a low patient and/or event number impedes strong conclusions from low prevalence mutations outcome association analyses in the medium-sized cohorts that are available, including ours [21,22]. Yet, our cohort had similar clinical and genetic features as comparable cohorts. Indeed, to mitigate the uncertainty caused by the sample size, we only tested associations with those mutated genes that were present in at least 10 patients with a PFS event.

Furthermore, poor outcome of patients with *TP53*-mutated tumors has been reported in multiple studies [8,10,11], even after correcting for HPV-status. However, each of these studies selected a different subset of *TP53* SPMs, as identified by different algorithms aimed at discriminating harmful from benign *TP53* SPMs. This difference in the classification of *TP53* mutations hampers comparison between studies. One should also note that it remains uncertain whether HPV-negative tumors classified as *TP53*-wildtype are truly *TP53*-wildtype as multi-codon deletions are difficult to detect with short-read sequencing technologies. 

The discrepancies between studies underscore the challenges of HNSCC prognostic biomarker research that lag behind those of other cancer types [13,35]. Owing to a high heterogeneity of the disease, it is difficult to obtain large cohorts of patients treated with the same modality and with homogenous tumors (in terms of stage and site). Comparing oral with pharyngeal HNSCC, our previous study [15] pointed to the presence of discrete genomic features in each site. Patient outcome parameters will be also largely affected by the different treatment options in a tumor biology and genomics dependent manner, and this highlights the need for treatment uniformity in such cohorts. We therefore opted for a homogenous cohort of patients to assess the impact of frequent HNSCC gene mutations.

The association of co-occurring *CCND1* and *CDKN2A* mutations with short PFS is remarkable, given the similar role in cellular biology. The protein product of *CCND1*, cyclin D1, forms a complex with cyclin-dependent kinases (CDKs) *CDK4* or *CDK6*. This complex promotes cell cycle progression through the G1/S checkpoint but can be inhibited by the protein product of *CDKN2A*, p16. As either *CCND1* amplification or *CDKN2A* loss could promote cell cycle progression, these mutations seem redundant at first sight [36]. The high prevalence of co-occurring *CCND1* and *CDKN2A* mutations in HNSCC does however suggest an additional growth advantage [36,37] and is supported by our findings and in vitro and in vivo data of others [38,39]. Supported by immunohistochemistry, FISH or RT–PCR, four earlier studies reported that the co-occurrence of *CCND1* overexpression or gain and the *CDKN2A* loss of expression, under-expression, or deletion marks a specific group in HNSCC with poor prognosis [40,41,42,43]. This is consistent with our findings based on gene mutation analyses. The deletion of *CDKN2A* may influence the interaction opportunity of survivin with CDK4 and further supports cell proliferation driven by the cyclin D amplification [44]. Survivin also suppresses apoptosis and its overexpession has been shown to be associated with poor prognosis in surgically resected HNSCC and OSCC [45,46].

Strong genetic biomarkers can guide clinical decision-making in the future. Patients with a confirmed poor prognosis may be eligible for a different, intensified, or targeted treatment and/or may benefit from increased surveillance. Genetic biomarkers can also provide clues for such alternative treatments and administration of targeted agents. *CCND1* overexpression due to amplification and its cellular activity or loss of *CDKN2A* is for example targetable with *CDK4/6* inhibitors [4,47,48]. One such inhibitor, palbociclib, is currently being tested for HNSCC [49].

## 4. Materials and Methods 

### 4.1. Patients

Pretreatment HNSCC samples were collected by biopsy and fresh frozen for biobanking; they were available for retrospective analyses. All patients gave their informed consent. The study was conducted in accordance with the Declaration of Helsinki and was approved by the Ethics Committee, the Institutional Review Board of the Netherlands Cancer Institute (P05MD1). Samples were obtained from 77 patients with oro- and hypopharyngeal carcinoma and treated with cisplatin based chemoradiotherapy at our institute between 2001 and 2010. Biopsy material with at least 50% tumor cells, as determined on H&E (haematoxylin and eosin stained) sections, was processed for DNA extraction. Matched normal samples were unavailable for the majority of tumors, and genomic analyses were therefore performed on tumor samples only. Patient cohort and tumor characteristics are described in Table 1.

### 4.2. Sequencing and Bioinformatics Protocol

Details of the DNA and RNA sequencing and bioinformatics protocols are specified in the Supplementary Methods [50,51,52,53,54,55,56,57,58,59]. In short, we performed target capture DNA sequencing of 556 human genes (Supplementary Materials: Table S2). HPV gene baits, to capture HPV DNA in the samples, were included in order to determine HPV status. We removed DNA sequence variants that were in any of three public SNP databases [54,55,56] and classified the remaining non-silent variants as SPMs. Homozygous deletions and focal amplifications were detected using the R package PureCN [57]. We used PureCN’s ploidy estimates as a chromosomal instability (CIN) marker. These estimates were rounded to the nearest integer and values not equaling 2 were classified as CIN+ (presence of CIN).

### 4.3. “HNSCC Driver Genes”

We used the “HNSCC driver genes” published by Iorio et al. that were identified by a large cohort of HNSCC. All HNSCC patients in this study underwent surgical resection and thus were not treated with definitive chemoradiotherapy [4]. A total of 168 “HNSCC driver genes” were identified by algorithms that select genes with more non-silent SPMs than expected by chance given various background mutation rates and processes (Supplementary Materials: Table S4). An additional 25 genes were selected from 33 regions identified as “drivers” by Iorio et al., by an analysis that selects regions with recurrent CNAs (Supplementary Materials: Table S5). We selected all genes that reside within these regions and that were annotated in the Cancer Gene Census [60] (Supplementary Materials: Table S5). A total of 27 out of the 168 SPM affected genes and 11 out of the 25 genes with recurrent CNAs (Supplementary Materials: Tables S4 and S5) were captured in our targeted sequencing panel (Supplementary Materials: Table S2). These amounted to 35 unique “HNSCC driver genes”, as three genes (*CDKN2A*, *EGFR*, and *NOTCH1*) were identified by both SPMs and CNAs.

### 4.4. Statistical Methods

Categorical and numerical variables were compared between groups with Fisher’s exact test and the Wilcoxon rank–sum test, respectively. Median follow-up time was derived from a reverse Kaplan–Meier analysis [61]. Except for in-figure log–rank test *p*-values, all reported survival analyses were obtained with multivariable Cox proportional hazards models that were used to test for associations between the selected genomic factors and PFS. Each model included the genomic factor, HPV status, and tumor site. HPV status and tumor site were included since they are strong prognostic factors in advanced HNSCC and were also prognostic in our cohort (Table 1 and Supplementary Materials: Table S1). All statistical analyses were performed in the R environment for statistical computing.

## 5. Conclusions

Our study shows that mutational burden estimates, CIN/aneuploidy markers, and co-occurring *CCND1* and *CDKN2A* mutations in advanced stage oro- and hypopharyngeal HNSCC are associated with patient outcomes. This highlights their potential as prognostic factors in advanced stage HNSCC patients treated with chemoradiotherapy and may have implications for targeted therapies or immunotherapies.

## Figures and Tables

**Table 1:** Patient and tumor characteristics. Follow-up and progression free survival (PFS) times in years.

Variable	All Patients	HPV-Negative	HPV-Positive	*p*-Value
	*N* = 77	*N* = 56	*N* = 21	
	*N* (%)	*N* (%)	*N* (%)	
*Gender*				
Male	22 (29)	17 (30)	5 (24)	0.778
Female	55 (71)	39 (70)	16 (76)	
*Tumor site*				
Hypopharynx	28 (36)	28 (50)	0 (0)	<0.001
Oropharynx	49 (64)	28 (50)	21 (100)	
*Disease stage*				
III	11 (14)	9 (16)	2 (10)	0.717
IV	66 (86)	47 (84)	19 (90)	
*cT classification*				
T1-T3	49 (64)	33 (59)	16 (76)	0.192
T4	28 (36)	23 (41)	5 (24)	
*cN classification*				
N0-N2a	24 (31)	20 (36)	4 (19)	0.181
N2b-N3	53 (69)	36 (64)	17 (81)	
*Smoker*				
Former	20 (26)	12 (21)	8 (38)	<0.001
Never	7 (9)	1 (2)	6 (29)	
Unknown	2 (3)	2 (4)	0 (0)	
Yes	48 (62)	41 (73)	7 (33)	
*Alcoholic consumption*				
Former alcoholic	13 (17)	13 (23)	0 (0)	0.005
Never	10 (13)	4 (7)	6 (29)	
Unknown	2 (3)	2 (4)	0 (0)	
Yes	52 (68)	37 (66)	15 (71)	
*Median age at diagnosis (range)*	58 (27–78)	58.5 (37–78)	57 (27–77)	0.175
*Median follow-up (95% CI)*	8.2 (6.7–10.5)	9.3 (7.8–NA)	6.6 (5.9–10.2)	0.113
*Number of PFS events (%)*	47 (61)	43 (77)	4 (19)	<0.001
*Median PFS (95% CI)*	5.8 (4.5–8.9)	4.5 (1.8–6.2)	NA	<0.001

**Table 2:** Prevalence of selected driver gene mutations and association with progression free survival (PFS).

Gene	Mutation ^a^	Prevalence				Survival Analysis ^b^	
		All Patients	HPV-Negative	HPV-Positive	*p*-Value	PFS	
		*N* = 77	*N* = 56	*N* = 21			
		*N* (%)	*N* (%)	*N* (%)		HR	*p*-Value
*TP53*	SPM	48 (62)	47 (84)	1 (5)	<0.001	0.87	0.753
*CCND1*	A	39 (51)	39 (70)	0 (0)	<0.001	2.1	0.079
*CDKN2A*	SPM or D	23 (30)	23 (41)	0 (0)	<0.001	1.17	0.663
*PIK3CA*	SPM	16 (21)	10 (18)	6 (29)	0.35	1.16	0.732

^a^ SPM = Somatic Point Mutation, A = Amplification, D = Deletion. ^b^ Multivariable Cox model with the additional independent variables HPV status and tumor site.

**Table 3:** The association of mutational burden (total number of somatic point mutations) and presence of the chromosomal instability marker (CIN) with progression free survival (PFS).

Variable	Prevalence				Survival Analysis ^c^	
	All Patients	HPV-Negative	HPV-Positive	*p*-Value	PFS	
	*N* = 77	*N* = 56	*N* = 21			
	*N* (%)	*N* (%)	*N* (%)		HR	*p*-Value
*Mutational burden* ^a^						
Per tumor	5 (0–16)	5 (1–14)	2 (0–16)	0.006	1.08	0.197
*Mutational burden groups* ^b^						
High	31 (40)	26 (46)	5 (24)	0.116	1.87	0.089
Low	46 (60)	30 (54)	16 (76)			
*CIN* ^b^						
Positive	32 (42)	30 (54)	2 (10)	<0.001	2.3	0.023
Negative	45 (58)	26 (46)	19 (90)			

^a^ Reports the median, range, and Wilcoxon rank–sum test p-value. ^b^ Reports the absolute number of tumors, percentage, and Fisher’s exact test p-value. ^c^ Multivariable Cox model with HPV status and tumor site as additional independent variables.

## Supplementary Materials

The following are available online at https://www.mdpi.com/2072-6694/11/4/445/s1: Figure S1. Progression free survival (PFS) stratified by HPV status and tumor site; Figure S2. Tumor mutational burden; Figure S3. Progression free survival (PFS) according to genetic alterations in *TP53*, *PIK3CA*, *CCND1*, and *CDKN2A*; Figure S4. Mutational burden and chromosomal instability features in samples according to *CCND1* and *CDKN2A* mutation co-occurrence; Table S1. Patient and tumor characteristics of HPV-negative tumor samples, stratified by tumor site; Table S2. List of targeted genes in DNA sequencing capture set; Table S3. Classification of the identified somatic point mutations; Table S4. List of "HNSCC driver genes"; Table S5. List of "HNSCC driver regions"; Table S6. Frequency table of co-occurring gene mutations, Supplementary Methods.
