# Supplementary material for: Genetic Factors Associated with a Poor Outcome in Head and Neck Cancer Patients Receiving Definitive Chemoradiotherapy
Source: Cancers (Basel). 2019 Mar 29;11(4):445. doi: 10.3390/cancers11040445 (PMC6521057; doi:10.3390/cancers11040445)
Supplement: Supplementary file 1 [file cancers-11-00445-s001.zip › Prognostic genomic factor Sup files/Prognostic_Genomic_Factors_Sup_Figures_v4.pptx]

## Slide 1
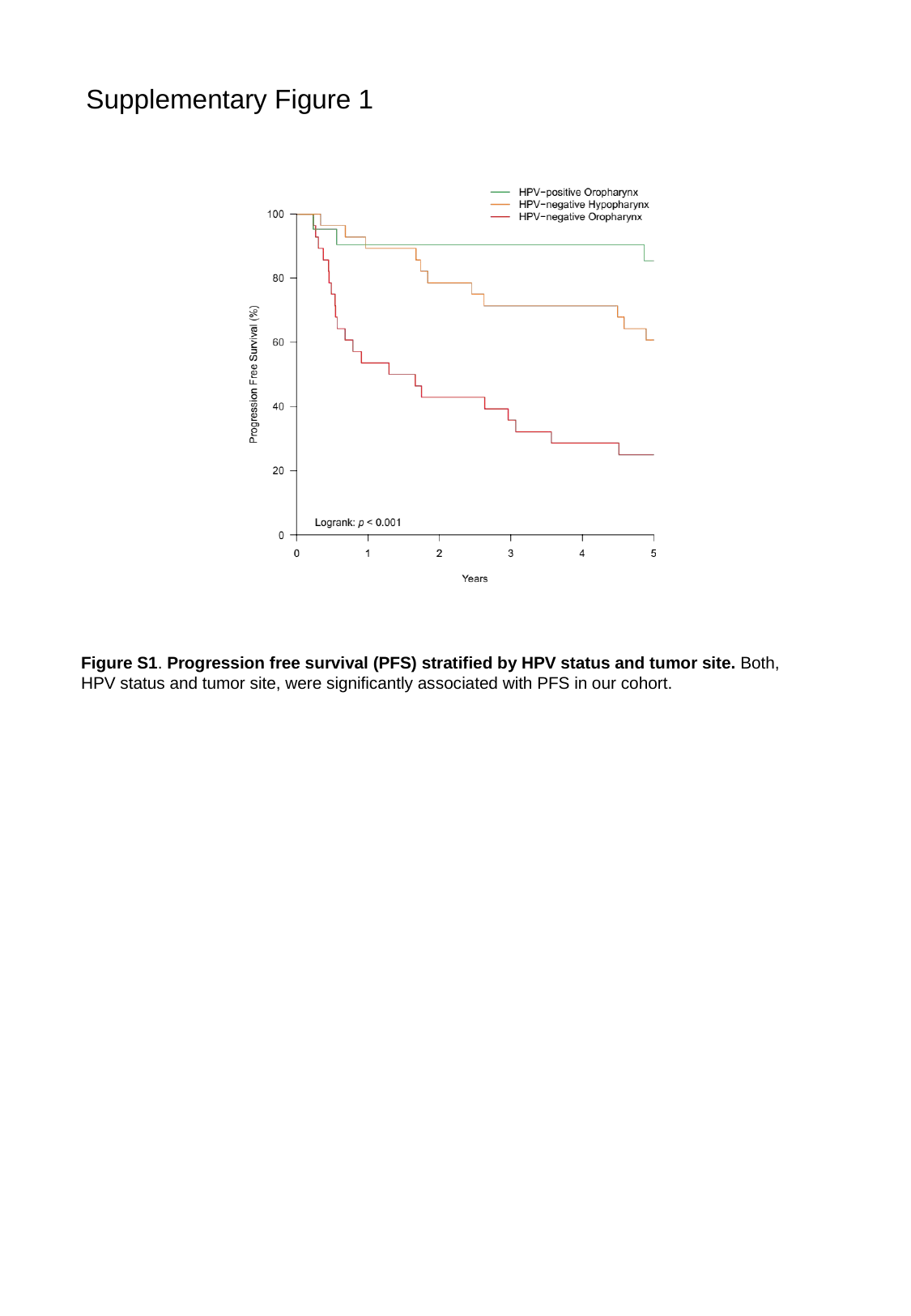

Supplementary Figure 1
Figure S1. Progression free survival (PFS) stratified by HPV status and tumor site. Both, HPV status and tumor site, were significantly associated with PFS in our cohort.

## Slide 2
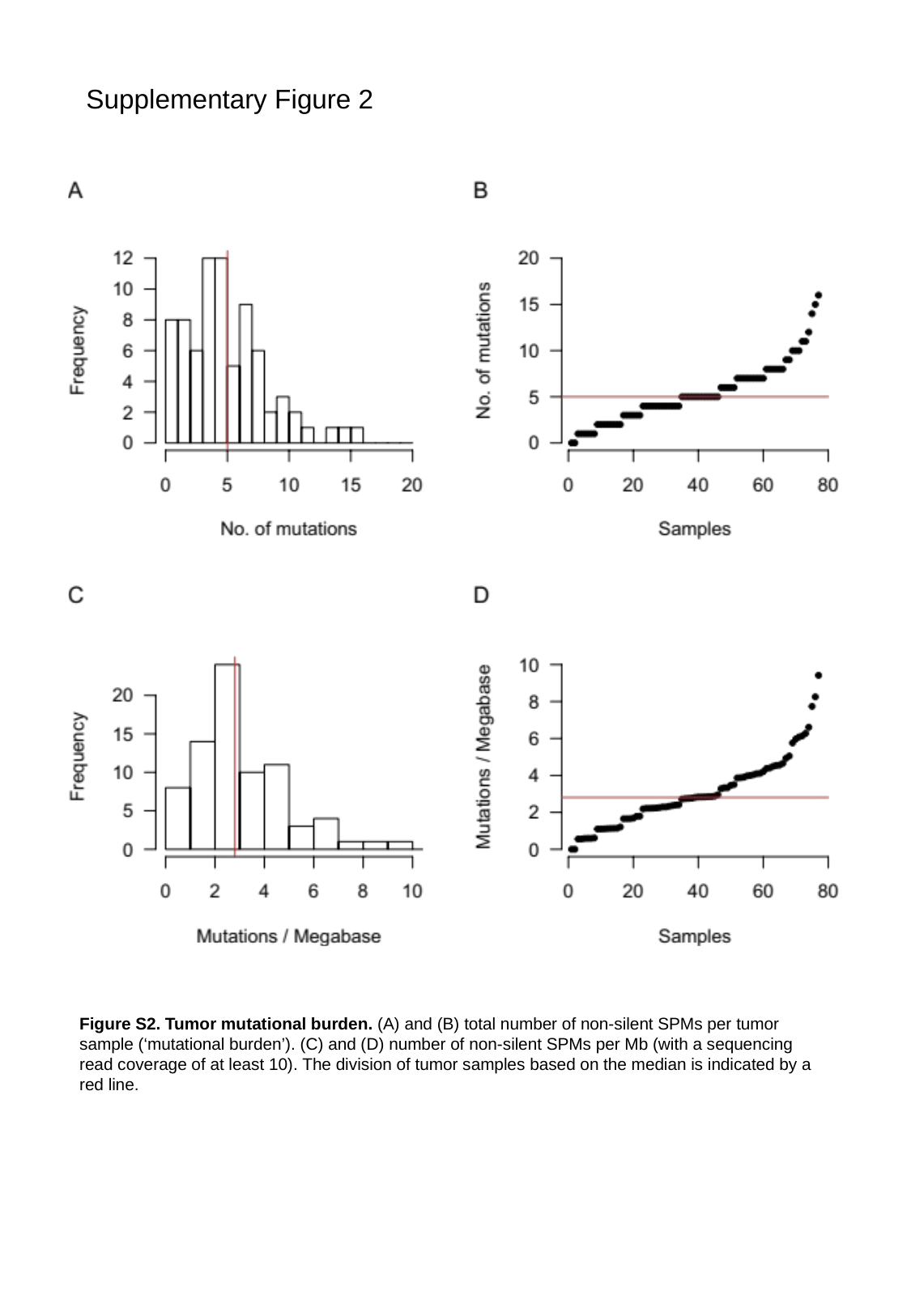

Supplementary Figure 2
Figure S2. Tumor mutational burden. (A) and (B) total number of non-silent SPMs per tumor sample (‘mutational burden’). (C) and (D) number of non-silent SPMs per Mb (with a sequencing read coverage of at least 10). The division of tumor samples based on the median is indicated by a red line.

## Slide 3
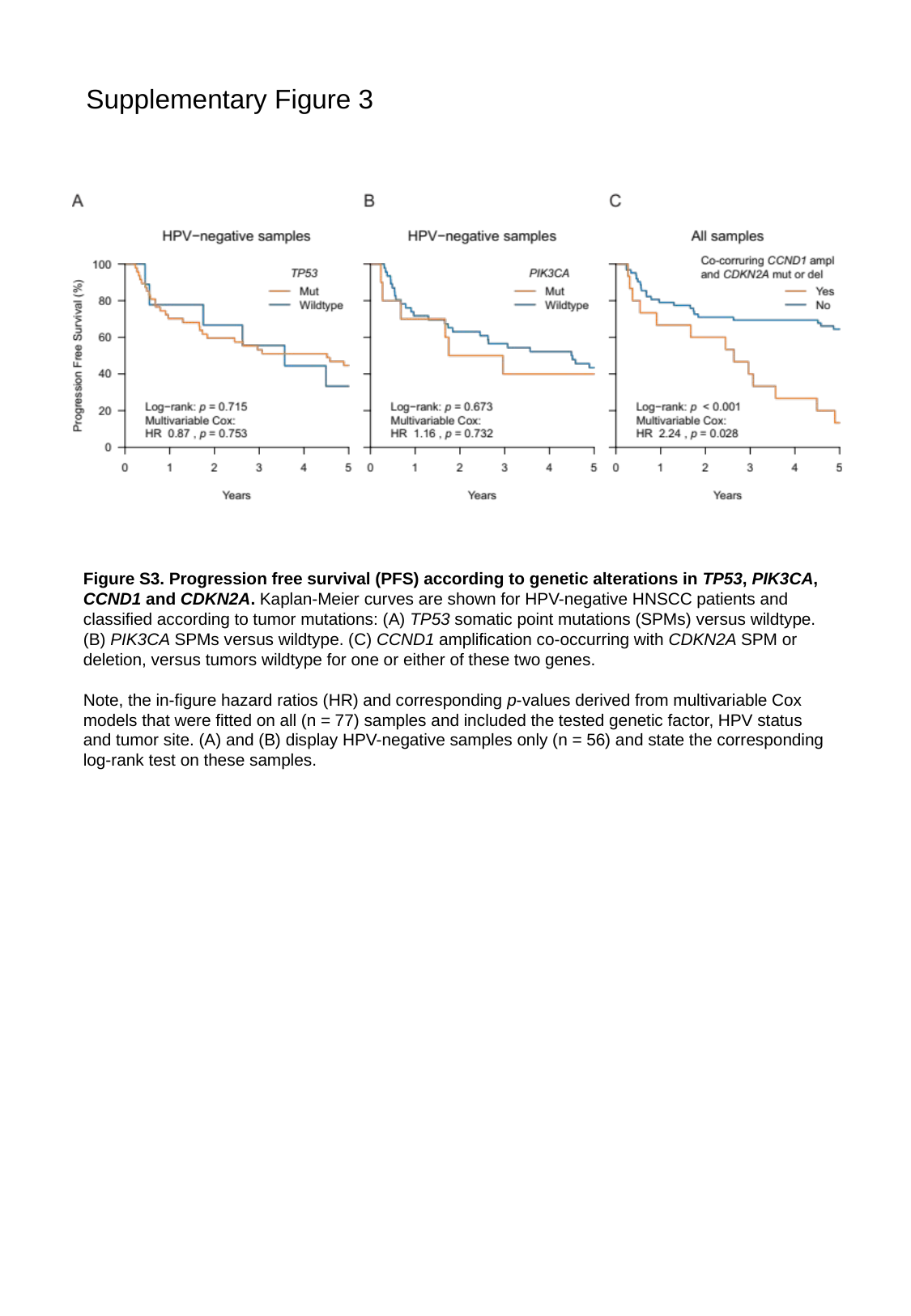

Supplementary Figure 3
Figure S3. Progression free survival (PFS) according to genetic alterations in TP53, PIK3CA, CCND1 and CDKN2A. Kaplan-Meier curves are shown for HPV-negative HNSCC patients and classified according to tumor mutations: (A) TP53 somatic point mutations (SPMs) versus wildtype. (B) PIK3CA SPMs versus wildtype. (C) CCND1 amplification co-occurring with CDKN2A SPM or deletion, versus tumors wildtype for one or either of these two genes.
Note, the in-figure hazard ratios (HR) and corresponding p-values derived from multivariable Cox models that were fitted on all (n = 77) samples and included the tested genetic factor, HPV status and tumor site. (A) and (B) display HPV-negative samples only (n = 56) and state the corresponding log-rank test on these samples.

## Slide 4
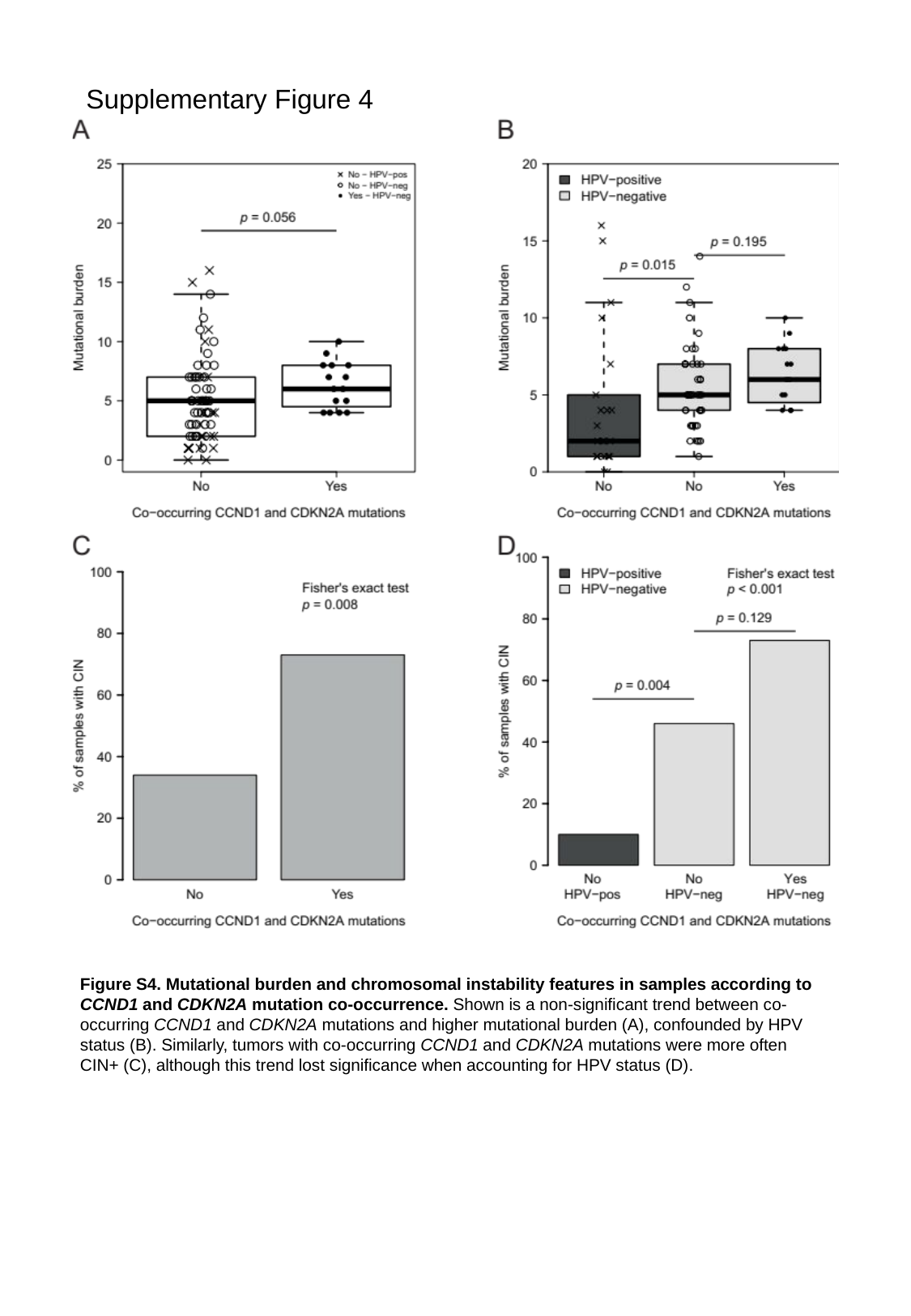

Supplementary Figure 4
Figure S4. Mutational burden and chromosomal instability features in samples according to CCND1 and CDKN2A mutation co-occurrence. Shown is a non-significant trend between co-occurring CCND1 and CDKN2A mutations and higher mutational burden (A), confounded by HPV status (B). Similarly, tumors with co-occurring CCND1 and CDKN2A mutations were more often CIN+ (C), although this trend lost significance when accounting for HPV status (D).
